# The Generation of Virtual Defenders

**DOI:** 10.1016/j.isci.2020.101118

**Published:** 2020-05-03

**Authors:** Michelle Muzzio

## Main Text

**Figure:**
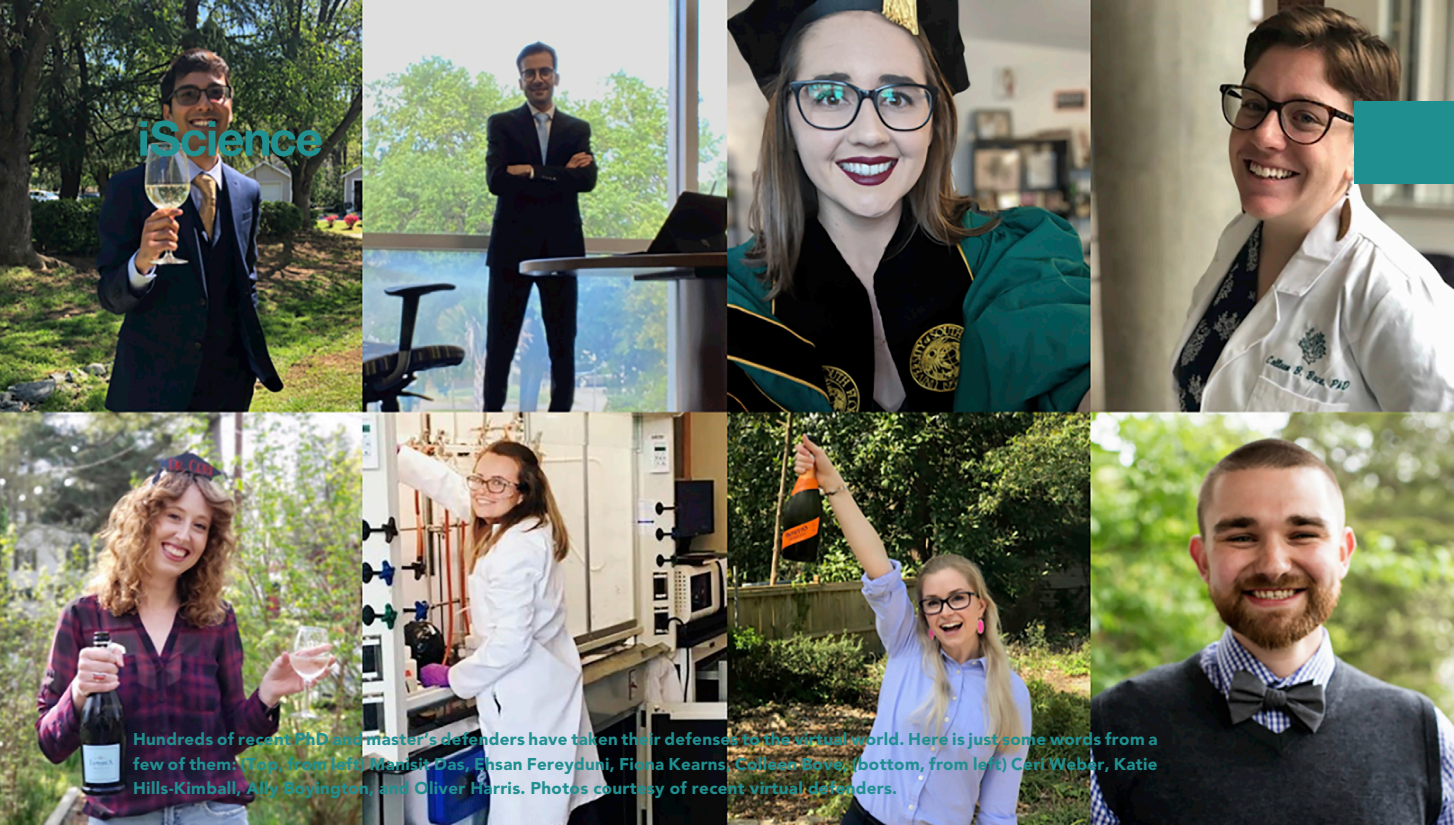
Hundreds of recent PhD and master's defenders have taken their defenses to the virtual world. Here is just some words from a few of them: (Top, from left) Manisit Das, Ehsan Fereyduni, Fiona Kearns, Colleen Bove, (bottom, from left) Ceri Weber, Katie Hills-Kimball, Ally Boyington, and Oliver Harris. Photos courtesy of recent virtual defenders.

Thesis defenses, a cornerstone of any advanced degree, are filled with emotions no matter what the circumstances. However, for hundreds of students planning to defend their master's or PhD theses in early 2020, another challenge was placed upon them: planning a *completely* virtual defense. Owing to COVID-19, social distancing requirements lead to thesis deliberations behind computer screens, champagne bottles to be popped outside at least six feet away from one or two close friends or family members, and more obvious technological concerns than trying to get a projector to work. Anxiety about thesis content became overwhelmed by sharing streaming links around the world or the rise of “zoom-bombers” finding their way in to wreak havoc by hacking into thesis defenses. However, with such anxiety, new avenues of science communication were also suddenly open. These recent PhDs had to very quickly alter presentations, consider the possibility of ***not*** hearing the audience laugh at their jokes, and think also about sharing their presentation with a wider, more interdisciplinary audience than they ever imagined within the walls of their own department.

Below, eight recent PhDs and two professors reflect on their experiences with virtual defenses. Some, such as Manisit Das and Ehsan Fereyduni used the virtual platform to open their defense around to world as far as India and Iran. Others, like Fiona Kearns and Colleen Bove, took this unforeseen event as an opportunity to take their science communication to the next level, in fields as wide as computational chemistry to coral reefs. Far too many, like Ceri Weber faced the unprecedented events of “zoom-bombing” but took a stand on social media and beyond to make sure that these assaults would not happen to any other hopeful virtual defenders. Channeling the voice of many graduate students, Katie Hills-Kimball, Ally Boyington, and Oliver Harris reflect on the grief felt, the pressure of defending in such an uncertain time, and the sometimes funny challenges that holding your defense in your living room may bring. Additionally, Prof. Leaf Huang, who has been a professor for over forty years, and Prof. Ou Chen, who is now just having his first cohort of PhD students ever graduate, reflect on how virtual defenses have affected them.

### First Impression

#### What Was Your First Reaction to Being Told You or Your Student Had to Defend Virtually?

I was definitely initially upset.

**Ally (Emory University, Organic Chemistry, Advisor: Nathan Jui):** I was definitely initially upset. This is a milestone that I had been envisioning for so long, and it felt like it was being taken away from me. I was also upset that family and friends who were supposed to travel to attend the in-person defense were not going to be able to.

**Ehsan (University of Florida, Organic Chemistry, Advisor: Alexander J. Grenning):** I was 4 days away from my defense date when I was informed that I should do my defense completely online. I was not too worried about the format of the presentation, but about the unpredictable events like Wi-Fi disconnection and power outages.

**Colleen (UNC Chapel Hill, Environment, Ecology, and Energy Program, Advisor: Karl Castillo):** I am a pretty nervous public speaker but filling the room with friendly faces always made it easier so that was tough to process. I also only had about 3 days to make sure my presentation was more friendly for digital-only viewing, which was stressful.

**Fiona (University of South Florida, Computational Chemistry, Advisor: Henry Lee Woodcock):** The PhD experience is very stressful. Understandably, I have been daydreaming about this day for years. Grief turned to guilt when my husband, a doctor, would come home day after day reporting more “COVID-likely” patients in his hospital. Now I felt sad and guilty for feeling sad because there were people out there truly suffering with the disease and the “stay home” measurements were meant to save lives. I decided if I had to have my defense online, I would drum up as many viewers, from as many different backgrounds, as possible. I wanted to tackle one last PhD science communication challenge!

**Katie (Brown University, Chemistry, Advisor: Ou Chen):** I was really upset when I found out that I would have to defend virtually. I continuously pictured the moment where I would get my turn to stand up in front of all the people that supported, mentored, and cared about me and feel proud and joyful for having “made it.”

**Manisit (UNC Chapel Hill, Pharmaceutical Sciences, Advisor: Leaf Huang):** Originally, I intended to have a traditional in-person defense, coupled with a streaming link because my parents and friends from India and other parts of the world couldn't make it to the defense physically. I was initially disappointed when I realized I had to defend virtually wholly, but in the end, many of my friends, colleagues, and folks who I interact with via social media joined and supported my defense.

**Leaf (Professor, UNC Chapel Hill):** I have been a professor for 43 years, and this is the first time that my own student had to do the defense in the virtual world. I was somewhat uncertain if the attendance will be good, but Manisit had over 70 people in attendance. I also found the tech support from our school was excellent.

**Ou (Professor, Brown University):** I feel disappointed and especially sorry for my students who will defend virtually, like Katie, who had family and friends traveling in for the defense. My immediate action was to check with my students who plan to graduate this spring (my first batch of PhD students in my career) and see if they still want to stick with their defense date and graduation time.

### Methods

#### What Technical Challenges Arose as You Planned Your Virtual Defense? What Platforms Were in Place to Support You as You Planned Your Virtual Defense?

**Ally:** I was actually most nervous about the fact that I live in an apartment so I can't always be in control of the noise level. The apartment is a 100-year-old house with creaky wood floors, and I'm on the first floor, so I actually sent an email to my neighbors asking them to be extra-conscientious about loud shoes or moving furniture during the hour of my defense.

**Katie:** My department gave instructions how to download Zoom and set up an event, but there were concerns about “zoom-bombing.” To prevent this, I set my advisor as a co-host and followed guidelines online to secure my defense.

**Fiona:** I decided to use FacebookLive and YouTubeLive. I know my parents know how to use Facebook. YouTube is great because you then have the video saved, people can watch later, and (assuming you're happy with the defense) you have the video saved and can use it to help build a future teaching portfolio.

#### What Technical Challenge Arose during Your Virtual Defense and How Did You or Your Team Face Them?

My lab mates (who I designated as the co-hosts) were frantically trying to shut them [the zoom-bombers] down as I tried to present.

**Ceri (Duke University, Cell Biology, Advisor: Blanche Capel):** My defense was zoom-bombed. Initially, we thought it was just someone having trouble with the microphone, but everyone should have been muted. The audio disruptions kept happening during my advisor's introduction too. Once I began to speak, the individuals started actually saying things to me like repeating words I had just said, playing music, and eventually threatening me. My lab mates (who I designated as the co-hosts) were frantically trying to shut them [the zoom-bombers] down as I tried to present. They locked the room so no one could enter and had to manually eliminate individuals with suspicious names or who kept speaking. After about 10 min, the disruptions stopped, and I was able to finish presenting undisturbed. My lab mates are heroes!

**Colleen:** I was not able to mute participants who did not mute themselves, and I think that was distracting for people signing in. Questions were a little awkward, but I had someone read out questions people typed into the chat function for me to answer, which worked well enough.

These three are highly intelligent people, but they are not computational chemists, so there were still some gaps I needed to close. Slide by slide I asked myself: is there some way I could quickly help them understand what I'm trying to say here?

**Manisit:** I assigned one of my friends as a co-host for the meeting in case someone inadvertently unmuted their microphone and to help with reading questions or comments from the chat. Zoom also has a breakout room feature, which I used to seamlessly transition from public to the private part of the defense, and eventually to walk out while my committee members discussed in my absence.

### Language

#### What Considerations Did You Take to Alter Your Presentation Considering that There Could Potentially Be Many Backgrounds Present in the Audience?

**Fiona:** To construct the presentation, I thought a lot about my parents and my husband. My mother has been a nurse for over 35 years, my father has a bachelor's degree in oceanography and has taught science at many grade levels, my husband is a medical doctor. Out of all of my audience members, I wanted to talk to them. They have helped me get to where I am, they deserve to know what I have made from their love and support. These three are highly intelligent people, but they are not computational chemists, so there were still some gaps I needed to close. Slide by slide I asked myself: is there some way I could quickly help them understand what I'm trying to say here?

**Ally:** I ended up not changing it too much because I wanted it to be as much of a normal defense as possible, but I wanted to make it as clear as possible to non-scientists and non-organic chemists what the over-arching goal of my work has been. No one else in my family is a scientist so I really wanted my passion for my work to come across.

**Katie:** I worked on my introduction a bit more since I could potentially have more people that aren't a part of my department at the defense.

**Ceri:** I wanted to create a presentation that would be engaging, easy to follow, and accessible to my family, friends, and honestly anyone who wanted to watch. I tried to maximize visuals and animations, so that there was always something happening on the screen for my audience to watch and pay attention to. Instead of getting into the details of an experiment, I wanted to focus on why we did it, what it told us, and what that led us to do next.

**Colleen:** After we opened it up to more people, I did decide to make a few more adjustments to how I described my research and the implications. Luckily, studying coral reefs does have the advantage of being a topic a lot of people have at least some experience with!

**Manisit**: I tried to emphasize the big picture and, while I went deep into the data, also explained what that results meant in the scope of the work and why we should care about it. There were a few features available on Zoom to interact with the audience, such as conducting polls, although I haven't used it.

### Presentation

#### What Was Different about Presenting for a Completely Online Audience Compared with Traditional In-Person Presentations?

**Oliver (Drexel University, Engineering, Advisor: Maureen Tang)**: Another challenge is that attention spans might be stretched more thinly when attending a virtual thesis defense. As a presenter the body language of audience members is often how I gauge how engaged my audience is. When presenting virtually, it is impossible to make the same sort of determinations about your audience's interest level.

**Ally:** My energy had to be much higher so that the talk was still engaging. It was great to get so many questions from people after the talk from all different parts of the country and all different fields. I also appreciated everyone who sent me a message or tweet afterward to congratulate me!

**Colleen:** I like to make people laugh in my presentation, it keeps my nerves down and lets me know people are with me, but this was missing from my virtual audience. I also had to make more of an effort to describe the content on my slides without just pointing like I would normally.

**Fiona:** To regain some interaction, I asked my viewers on Facebook to post selfies and “react” to the stream, to help me have some element of feedback. But still, the interaction between presenter and audience is gone with an online defense. However, the one thing that was undoubtedly the same between traditional/physical presentations and the online presentation was the presentation jitters!

**Figure:**
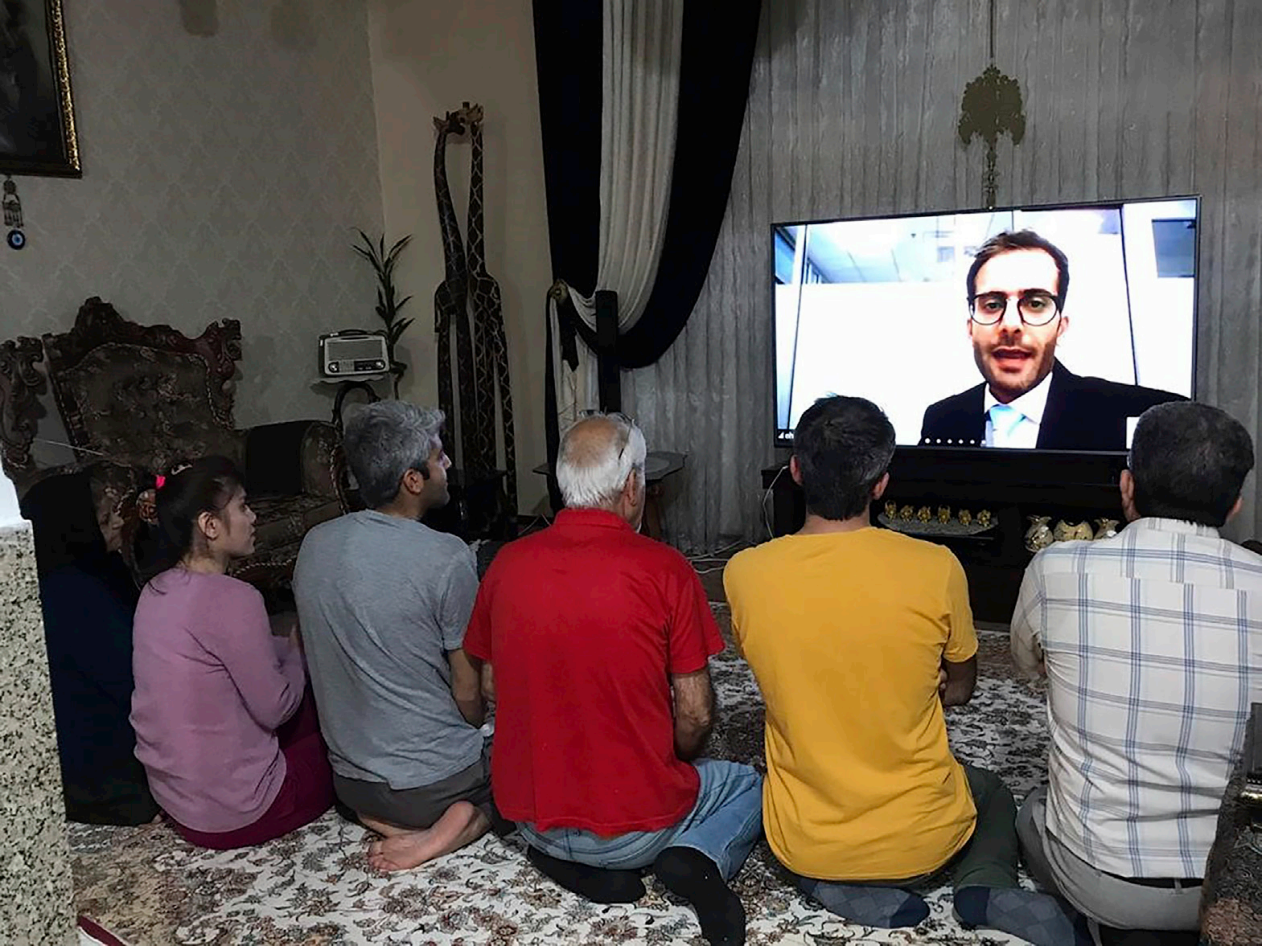
Fereyduni streamed his defense across the world. Here is a photo of his family in Iran watching him embrace his milestone defense to become a PhD. Photo courtesy of Fereyduni.

### Future

#### Do You Think There Is a Future for Virtual Defenses?

**Fiona:** Maybe funding grants can be awarded to students who would like to stream their defense online. Additionally, like poster competitions or the 3MT competitions, maybe students can compete to have professional streaming support for their theses. Either way I think we should try to keep an element of online/virtual defenses because it is indeed wonderful to be connected over such a large distance; it only took a crisis to help us see that.

I think that virtual defenses, and maybe soon virtual conference presentations, will become a part of the next generation of open-science tools.

**Oliver:** I think that virtual defenses, and maybe soon virtual conference presentations, will become a part of the next generation of open-science tools. Additionally, I think moving forward, virtual presentations, seminars, will become more prevalent for economic reasons. The long-term effects of the outbreak are unknowable, but the economic impact is already clear, and universities, journals, funders, etc. will have to adapt to a new landscape of travel and science communication.

**Katie**: In the future, I think it would be cool to sort of combine the traditional defense with the virtual one so that the presenters could have their entire support system (near and far) present at the defense.

**Ehsan:** I think that it would be great if we could provide virtual access to the public portion of PhD defenses. Some people may have a valid argument that they have unpublished results that they don't like to be released publicly. But at the very least this can be an option for those who like to do it.

**Ceri:** Assuming the link is secure, people from around the world can watch a seminar and ask questions. After my defense, several experts in my field emailed my advisor to talk about my work, which was really exciting! Local schools and classes can join in too. Science should be shared with the community, and this is a great way to achieve that. Maybe we can start including more committee members outside of our departments and universities.

### Final Thoughts

#### How Are You Feeling about the Whole Process Now (by the way, Congratulations!)?

**Ally:** Now that it’s done I feel really positive about the way that it went. I'm am still bummed out that I didn't get to see family and friends in person that were supposed to travel, and disappointed that graduation is canceled, but it will definitely make a unique story to say that I defended my PhD from my living room.

**Fiona**: I travel often to Austria for research and I have lifelong friends there now. Because I moved to a fully online defense, my friends and advisors in Austria were able to watch it. One adviser, someone who has opened his lab to me—a foreigner—with continuous, gracious, hospitality, emailed me after my defense: “*In these times of quarantine it was wonderful to be connected over this large distance*.” Science and technology can connect people, and what a better moment to share science than at the end of a master's or PhD degree.

**Ehsan**: We all probably have had experiences with the online meetings but delivering my whole 5-year PhD journey in a 1-hour presentation followed by almost 2-hour Q&A with my PhD committee members all completely online was a unique experience. I hope hiring managers and recruiters now and in the future have special considerations for this class that graduated in these unprecedented circumstances.

#### What Advice Would You Give to Students or Professors as They Prepare for the Virtual Defense Experience?

**Manisit:** I advise practicing the talk with colleagues and friends, because talking for a long time before a screen can be super awkward at times! If you can, also join the meeting using a secondary screen, and encourage people to keep their video on if they are comfortable; this can be a great way to substitute audience interaction. Recently, there had been issues of online harassment with publicly posted links, so definitely improve security so that the meeting can be conducted without interruptions while sharing good science with the world!

**Ou:** Based on my experience from teaching virtually, my suggestion would be slow down your presentation and maybe take a couple of short stops during the defense. Since now you are watching your screen but not your audience, and without any movements during the presentation, it could be easy to go faster and faster.

**Leaf:** I still think the defense should be done in person if possible, but if anyone cannot attend in person, virtual attendance will be an excellent alternative.

**Katie:** This has been a very emotional time around the world, and I think it is reasonable that focus cannot be solely given to thesis preparation considering the current events, and that should be taken into consideration and you should allow yourself to feel and be human.

**Colleen**: The advice I have for people preparing for something similar is go into it treating it as the same as the “in-person” mentally, because no matter how many people are actually in the room, it is still an *amazing accomplishment* and you should still be proud. I will say that the lack of an actual celebration was tough for me since I had always planned to celebrate with family, friends, and peers. Take time to process it all and it is okay to be a little disappointed, but at the end of the day it counts and is still a massive achievement!

